# Summer Lectures

**Published:** 1836

**Authors:** 


### Art. IV.—Summer Lectures in the Cincinnati College.

Lectures on the various branches of medicine were commenced about the 1st of April in the Institution above mentioned. There have been eighteen lectures, accompanied by as many examinations, delivered weekly, to a respectable class of students. The course will continue until the month of October. All the lecturers, with one exception, belong to the Medical Faculty. They are arranged in two divisions, for the better accommodation of the students. Lectures on Theory and Practice, on Midwifery, on Chemistry and on Materia Medica will be commenced about the first of August; the month of July being a vacation. Students who design spending the winter in the city would do well to attend them as they would then be the better prepared for the winter course. W. W.

